# Electrodeposition of iron from 1-ethyl-3-methylimidazolium trifluoromethanesulfonate and reverse microemulsions of Triton X-100

**DOI:** 10.1098/rsos.230632

**Published:** 2024-05-15

**Authors:** Nazifa Tabassum, Shimul Saha, Md. Mominul Islam, Md. Abu Bin Hasan Susan

**Affiliations:** ^1^ Department of Chemistry, University of Dhaka, Dhaka 1000, Bangladesh; ^2^ Department of Chemistry, Bangladesh University of Engineering and Technology, Dhaka 1000, Bangladesh; ^3^ Dhaka University Nanotechnology Center (DUNC), University of Dhaka, Dhaka 1000, Bangladesh

**Keywords:** iron, electrodeposition, morphology, ionic liquid, reverse microemulsion

## Abstract

Electrodeposition of iron (Fe) was investigated in three different media, namely a hydrophilic ionic liquid (IL), 1-ethyl-3-methylimidazolium trifluoromethanesulfonate, conventional reverse microemulsion (RME)/reverse micellar solution, and IL-based RME of a non-ionic surfactant, Triton X-100, with a view to electrodepositing iron with desired morphology. Electrochemical behaviour of Fe^2+^ was studied using cyclic voltammetric technique with a copper electrode as the working electrode. Electrochemical reduction of Fe^2+^ in all the studied media was found to be an electrochemically irreversible, diffusion-controlled process. Successful potentiostatic electrodeposition of metallic iron was performed in all the studied media on copper substrate using bulk electrolysis method. The obtained iron electrodeposits were characterized using a scanning electron microscope and an X-ray diffractometer. The controlled diffusion of Fe^2+^ towards electrode surface in all the media resulted in the formation of nanoparticles of iron, but compact layers of granular nanoparticles could be achieved from both the conventional and IL-based RME systems. The IL-based microemulsions synergistically combined the advantageous features of both the IL and RME and showed promise for tuning the size, shape, and morphology of the electrodeposited iron.

## 1. Introduction

Iron (Fe) is one of the most naturally abundant, low-cost metals with versatile applications in daily life [1]. Multipurpose applications of metallic iron and its alloys include corrosion-resistant coatings [2,3], magnetic recording devices [4], transformer core materials [5], drug delivery [6], electroforming [7], catalysis [8,9], etc.

Electrodeposition is a cost-effective easy setup technique to obtain metals and alloys with desired size, shape and morphology [10,11]. Electrodeposition of iron has been widely attempted from aqueous media [12,13]. However, several drawbacks, such as formation of ferric hydroxide [14–16], limited electrochemical potential window [17,18], etc., have been reported during electrodeposition of iron from aqueous media. The earliest report on the interference of hydroxide formation at the time of electrodeposition was made by Kabanov *et al*. [14], Bonhoeffer & Jena [15] and later Bockris *et al*. [16]. Therefore, alternative non-aqueous systems such as organic solvents [19], ionic liquids (ILs) [11,20], deep eutectic mixtures [21], etc. were used for electrodeposition of metallic iron to overcome the limitations associated with aqueous systems. Among several non-aqueous media, ILs have been considered as an efficient medium for electrodeposition of metals and alloys due to their several advantageous properties such as wide electrochemical potential window [22], negligible hydrogen evolution [23], high thermal stability [24], etc. ILs have been reported to significantly affect the quality, crystalline size, and morphology of iron coatings [20].

It is worth mentioning that control over the microstructure and morphology of deposits is a highly focused area of current research for specific practical applications [25–27]. Materials with smaller particle size are reported to show different and unique properties from their bulk properties [28–30]. Thus, controlled regulation of the size and shape of iron particles can be useful to investigate the influence of reduced particle size on the intriguing features of iron coatings. In that case, utilization of surfactant based organized media, such as water-in-oil microemulsions based on reverse micelles, with controlled environment for electrodeposition can be very promising to obtain iron electrodeposits with tunable size, shape, and morphology by changing the composition of the medium. The core size of a reverse micellar system has been reported to affect the morphology and particle size of the depositing metals [31–35]. Besides conventional microemulsion based reverse micelles, reverse microemulsions (RMEs) with ILs can be very promising media for electrodeposition of metals and alloys due to synergistic advantageous properties of both RMEs and ILs. In RME systems with ILs, ILs are supposed to participate in both the formation of reverse micelles and strong double layer and/or layer by layer structure near the working electrode surface. Formation of unique layer structure near the working electrode surface by the ions of ILs is considered to play a vital role in achieving tunable size and morphology of the electrodeposited metals and alloys. Therefore, the combined effect of RME systems with ILs, double layer structure of ions of ILs near the working electrode surface and controlled environment of reverse micelles in the bulk can reveal itself as a potential medium to achieve electrodeposits with desired morphology. There are very few reports regarding electrodeposition of metals and alloys from ‘IL in water’ and ‘water in IL’ based microemulsion systems [36–39]. The terms ‘water in IL’ and ‘IL in water’ in these cases refer to two different types of microemulsions. In both cases, a hydrophobic IL has been used as the non-polar medium. In ‘IL in water’ microemulsion systems, water exists as the continuous phase with IL droplets as the dispersed phases inside the core of micelles and in ‘water in IL’ microemulsion systems, IL exists as the continuous phase with water droplets as the dispersed phases inside the core of reverse micelles. Fu *et al*. [37] reported electrodeposition of silver from ‘IL in water’ and ‘water in IL’ based microemulsions and electrodeposition of gold from ‘water in IL’ microemulsions. Serra *et al*. [38] reported electrodeposition of Co–Pt alloys from ‘IL in water’ microemulsions. Interestingly, all the researchers have used non-ionic surfactants to prepare IL in water or water in IL based microemulsion systems. Compared with ionic surfactants, non-ionic surfactants have many advantages as emulsifiers including less toxicity, stability over a wide pH range and less sensitivity to electrolytes. The great advantages of non-ionic surfactants are their lack of strong electrical charges, which enables them to be less affected by the ions in the electrolyte solution [40]. Therefore, IL-based RME systems composed of a non-ionic surfactant can be a more effective choice to study electrodeposition.

Considering the possibility of obtaining metallic iron with tunable size, shape and morphology, electrodeposition of iron has been attempted from a hydrophilic IL, 1-ethyl-3-methylimidazolium trifluoromethanesulfonate ([C_2_mim]CF_3_SO_3_), and RME systems of a non-ionic surfactant, Triton X-100 (TX-100), both with and without the IL. The ultimate goal of the present study is to reveal the morphological tunability with controlled size and shape of iron electrodeposits using the promising medium as a bath for electrodeposition.

## 2. Experimental

Ferrous sulfate (FeSO_4_) (Unichem Lab., USA, 99%) was used as the source of Fe^2+^ ions. Cyclohexane, TX-100, and 1-ethyl-3-methylimidazolium trifluoromethanesulfonate (Sigma Aldrich, USA, ≥98%) were used as received without further purification. Deionized water (conductivity 0.055 µS, BOE 8082063 UV/UF, BOECO, Germany) was used to prepare the conventional RME and for cleaning purposes. The conventional RME was prepared using TX-100, water as the polar phase and cyclohexane as the non-polar phase (table 1) [41]. While, a hydrophilic IL, [C_2_mim]CF_3_SO_3_, was used as the polar phase instead of water in the case of IL-based RME and the other components of IL-based RME were same as used for the conventional one (table 1). KClO_4_ (Wako Pure Chemical Industries, Japan, 99.5%) was added to the RMEs in the solid form as the supporting electrolyte. To prepare Fe^2+^ solutions, calculated amount of FeSO_4_ was dissolved in IL and RMEs followed by sonication using an ultrasonic bath (UNILAB UET-1080, USA) to ensure complete solubilization of the salt used.

**Table 1.** Compositions of the reverse micellar systems.

conventional reverse microemulsion				
components (wt%)	TX-100	cyclohexane	water	water to surfactant molar ratio, *W*_o_
	45	46	9	6.96
IL-based reverse microemulsion				
components (wt%)	TX-100	cyclohexane	water	IL to surfactant molar ratio, *R*_o_
	54	35	11	0.52

The electrochemical measurements and electrodeposition were carried out with a computer-controlled electrochemical analyser (CHI 660E and CHI 760E, CH Instruments, Inc., USA) using a conventional single-compartment three-electrode cell. Prior to each measurement, the solution was purged with nitrogen gas to maintain an inert atmosphere during the course of the measurement. A homemade copper disc electrode (*d* = 1 mm) was used as the working electrode during cyclic voltammetric measurements. The electrode was mechanically polished using 0.05 µm alumina paste (Buehler, USA) to obtain a uniform surface and then rinsed thoroughly with distilled water and dried. The dried electrode was then introduced into the cell filled with the test solution. A platinum (Pt) wire was used as the counter electrode. Ag/AgCl was used as the reference electrode in the conventional RMEs, while a Ag wire was used in the IL and IL-based RME systems. The Ag/AgCl reference electrode used in the conventional RMEs was found to be fairly stable during the course of the experiments which was judged by comparing the cyclic voltammograms performed at different time intervals. Metallic iron deposits were obtained on a copper substrate (*d* = 6 mm) with bulk electrolysis by applying constant potential using the same three-electrode single-compartment cell used for cyclic voltammetric measurements. After successful electrodeposition, the deposits were washed with deionized water and dried. The size, shape, and morphology of the deposits were studied by a computer-controlled scanning electron microscope (SEM) (ZEISS Sigma 300, Germany). The crystalline structure of the deposits was investigated by X-ray diffraction (XRD; Rigaku Smartlab SE, Japan).

## 3. Results and discussion

### 3.1. Electrodeposition of Fe in [C_2_mim]CF_3_SO_3_

**Figure 1.**
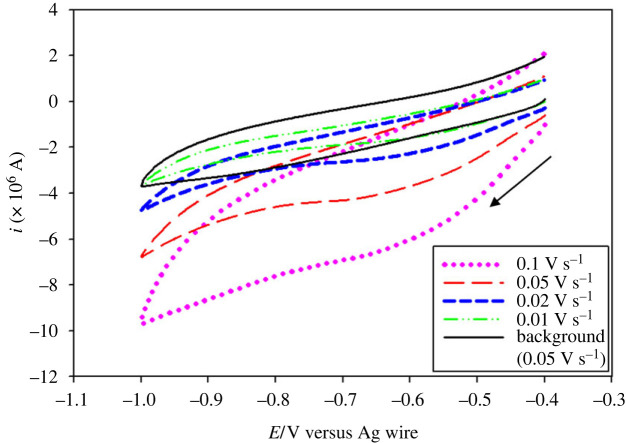
Cyclic voltammograms of 10 mM FeSO_4_ in [C_2_mim]CF_3_SO_3_ at different scan rates on a copper electrode at 30°C. The solid black line shows the cyclic voltammogram in neat [C_2_mim]CF_3_SO_3_.

[C_2_mim]CF_3_SO_3_, a hydrophilic IL, has been used in the present study as a medium for electrodeposition of Fe at ambient condition. Figure 1 shows the cyclic voltammograms of neat [C_2_mim]CF_3_SO_3_ at a scan rate (*v*) of 0.05 V s^−1^ and 10 mM FeSO_4_ in [C_2_mim]CF_3_SO_3_ at different *v* on a copper electrode at 30°C. The voltammograms were recorded by scanning the potential from open circuit potential (OCP) to −1 V in a forward scan followed by a reverse scan back to the OCP.

No Faradaic current was observed in the absence of FeSO_4_. However, in the presence of FeSO_4_ a cathodic peak current at around −0.62 V was observed, which can be due to the electroreduction of Fe^2+^ ions to metallic iron. In the anodic scan, no oxidation current peak was observed in the scanned potential range to indicate that the electrochemical reduction of Fe^2+^ is an irreversible process in the present experimental conditions. The observations are distinctly different for the electrochemical behaviour of Fe^2+^ in aqueous solution (electronic supplementary material, figure S1). Electrochemical reduction of Fe^2+^ to metallic iron was confirmed by constant potential deposition (*vide infra*).

**Figure 2.**
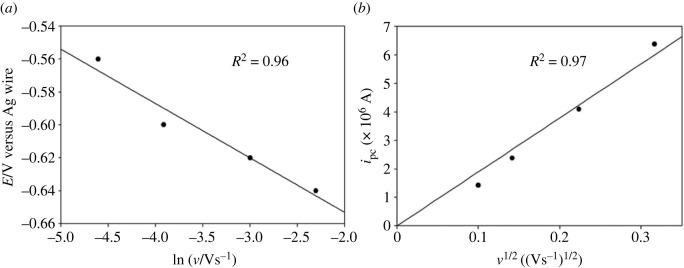
(*a*) Variation of cathodic peak potential with the logarithm of scan rate and (*b*) variation of cathodic peak current with the square root of scan rate for the cyclic voltammogram of 10 mM FeSO_4_ in [C_2_mim]CF_3_SO_3_ on a copper electrode at 30°C.

It is also evident from figure 1 that with increasing *v*, the cathodic peak potential *E*_pc_ shifts towards more negative value and the cathodic peak current *i*_pc_ increases gradually. Figure 2*a* shows the dependence of *E*_pc_ on the logarithm of *v* for the reduction of Fe^2+^ on a copper electrode. For an irreversible electrode reaction, the peak potential is known to be related to *v* by the following equation [42]:

3.1 $$E_{\mathrm{pc}}=E^{o\prime }-\frac{RT}{\alpha nF}\left[0.780+\ln\left(\frac{D^{1/2}}{k^{o}}\right)+\ln{\left(\frac{\alpha nF_{v}}{RT}\right)}^{1/2}\right],$$

where *E*°′ is the formal potential (V), *R* is the gas constant (erg mol^−1^ K^−1^), *T* is the absolute temperature (K), *F* is the Faraday constant (C), *D* is the diffusion coefficient of Fe^2+^ ions (cm^2^ s^−1^), *k*^o^ is the heterogeneous rate constant (cm s^−1^) and *n* is the number of electrons transferred in the redox process. The linear relationship between *E*_pc_ and ln(*ν*) also indicates that the electrode process for the reduction of Fe^2+^ in [C_2_mim]CF_3_SO_3_ is electrochemically irreversible. The increase of *i*_pc_ with the increase of *ν* as observed in figure 1 is expected for a diffusion-controlled process. The electrochemical behaviour of an irreversible diffusion-controlled process is best defined by the following equation [42]:

3.2 $$i_{\mathrm{pc}}=(2.99\times 10^{5})D^{1/2}\alpha^{1/2}CAn^{3/2}\nu^{1/2},$$

where *C* is the concentration (mol cm^−3^) and *A* is the electrode area (cm^2^). Figure 2*b* presents the *i*_pc_ versus *ν*^1/2^ plot, which shows a straight line passing through the origin indicating the electroreduction of Fe^2+^ is a diffusion-controlled process. Therefore, the electrochemical reduction of Fe^2+^ in [C_2_mim]CF_3_SO_3_ on copper electrode can be considered an irreversible diffusion-controlled process.

**Figure 3.**
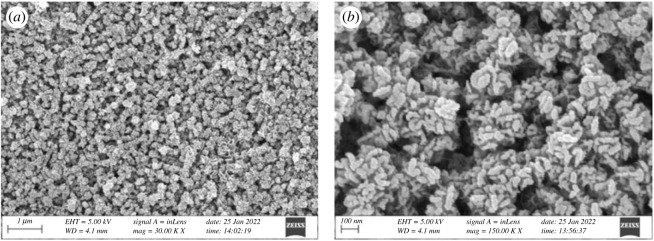
SEM images of electrodeposited iron obtained by potentiostatic electrolysis at −0.6 V for 1 h on a copper substrate in [C_2_mim]CF_3_SO_3_ containing 10 mM FeSO_4_. Magnification: (*a*) ×30 000; (*b*) ×150 000.

Electrodeposition of Fe^2+^ in [C_2_mim]CF_3_SO_3_ IL was successfully carried out at –0.6 V for 1 h on a copper substrate at 30°C. The obtained deposit was subjected to SEM analysis as shown in figure 3.

It is clear from the SEM images (figure 3) that the electrodeposit consists of particles in the nanometre range, which is not surprising as the electrodeposition of nanoparticles in ILs is well reported [43,44]. Whereas a random morphological bulk iron deposition is common during electrodeposition using an aqueous bath (electronic supplementary material, figure S2). Formation of unique charged double layer structure by the ions of IL near the electrode surface in IL media is considered to play the key role towards formation of nanoparticles during electrodeposition. However, the deposited layer was not compact probably due to the crowding of ions of IL during the electrodeposition process.

**Figure 4.**
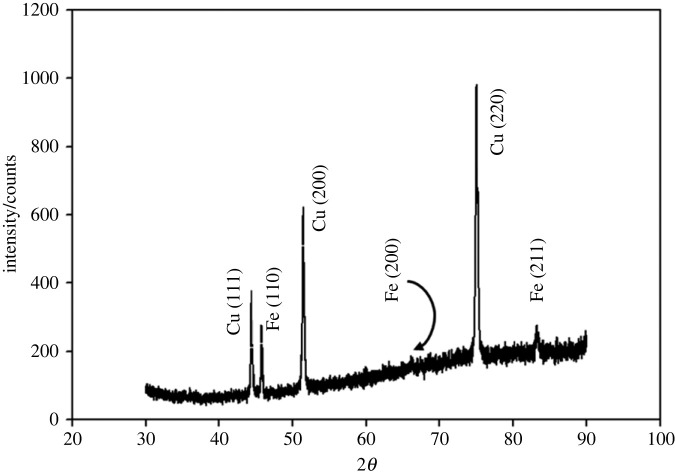
XRD pattern of iron deposit obtained by potentiostatic electrolysis at −0.6 V for 1 h on a copper substrate in [C_2_mim]CF_3_SO_3_ containing 10 mM FeSO_4_.

Electrodeposition of metallic iron on the copper substrate was confirmed by XRD analysis. Figure 4 shows the XRD pattern of the electrodeposited iron obtained by constant potential electrolysis at –0.6 V for 1 h in [C_2_mim]CF_3_SO_3_ on a copper substrate.

The crystal structure of the deposit was confirmed to be body centred cubic. The XRD pattern shows typical characteristic peaks for iron at 2*θ* = 45.78°, 66.50°, and 83.27°corresponding to the reflection planes of (110), (200), and (211), respectively [19,45]. The Fe (200) peak seems to be merged with the background noise, but the presence of this peak could be confirmed by experiments on similar systems (data not shown) consistent with the literature [19]. The peaks at 2*θ* = 44.40°, 51.43°, and 75.01°can be assigned to the reflection planes of (111), (200), and (220), respectively, for copper substrate. The crystallite sizes of iron electrodeposits were calculated using the Scherrer equation as follows:

3.3 $$\chi_{s}=\frac{0.9\lambda }{\mathrm{FWHM}\cos\theta },$$

where $\chi_{s}$ is the crystallite size, *λ* is the wavelength of X-rays (0.154 nm), FWHM is full width at half maximum and *θ* is the diffraction angle. For (110) and (211) planes, the crystallite size was found to be 77.2 nm and 28.4 nm, respectively. Therefore, electrodeposition of metallic iron in [C_2_mim]CF_3_SO_3_ can be possible with the formation of nano-sized particles at ambient condition. However, a combined effect of double layer structure of ions of IL near the working electrode surface along with the controlled environment of reverse micelles in the bulk solution could tune the morphology and particle size of the electrodeposited Fe with the formation of a compact deposition layer. Thus, further electrodeposition study of Fe^2+^ was attempted in conventional and [C_2_mim]CF_3_SO_3_ based RME media.

### 3.2. Electrodeposition of iron in conventional and ionic liquid-based reverse microemulsion systems

**Figure 5.**
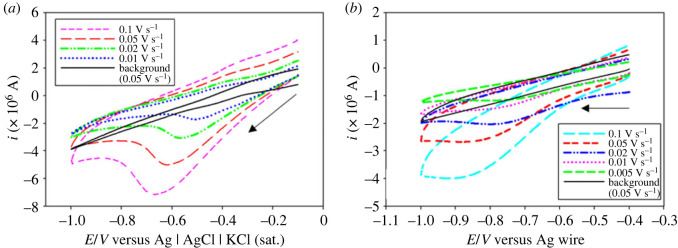
Cyclic voltammograms of (*a*) 3 mM FeSO_4_ in RME of TX-100 and (*b*) 20 mM FeSO_4_ in IL-based RME of TX-100 at different scan rates on a copper electrode at 30°C.

Electrodeposition of metals from surfactant-based organized media is reported to be quite different with controlled deposition rate [32,46]. In the present study, electrodeposition of iron in water-in-oil and IL-in-oil microemulsions of TX-100 was studied. Figure 5 represents the cyclic voltammograms of conventional microemulsions in the absence and presence of 3 mM FeSO_4_ and IL-based microemulsions in the absence and presence of 20 mM FeSO_4_ at various *v*s on copper electrode at 30°C. Due to the significant difference in solubility of FeSO_4_ in conventional microemulsion and IL-based microemulsions, the concentration of FeSO_4_ was chosen to ensure noticeable peak responses.

It is evident from figure 5*a*,*b* that the RME itself does not interfere with the reduction process in the potential range studied. In both cases, in the presence of FeSO_4_, a single reduction current peak was observed during the cathodic scan and no oxidation current peak was found in the anodic scan indicating irreversible nature of the electrochemical reduction of Fe^2+^ in both RME systems. The main differences for the electroreduction process of Fe^2+^ in conventional RME and IL-based RME systems were the reduction potential and the current. The electroreduction of Fe^2+^ in IL-based RME system occurs at a more negative potential compared to that of conventional RME, probably due to the strong complex formation of Fe^2+^ with the anion of the IL. Metal ions are known to form stable complexes with the anion of ILs [44,47]. Also, the electroreduction of Fe^2+^ in IL-based RME system gives less current compared to that of conventional RME due to higher viscosity of the IL-based RME system.

The shifting of the cathodic current peak towards more negative potential with increase of *v* is usual for an irreversible system. Moreover, the increase of the reduction current with increase of *v* indicates that the electrochemical reduction of Fe^2+^ in the present experimental condition is a diffusion-controlled process.

**Figure 6.**
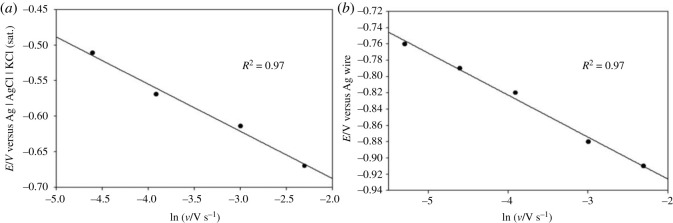
Variation of cathodic peak potential with the logarithm of scan rate for the cyclic voltammograms of (*a*) 3 mM FeSO_4_ in conventional RME of TX-100 and (*b*) 20 mM FeSO_4_ in IL-based RME of TX-100 on a copper electrode at 30°C.

**Figure 7.**
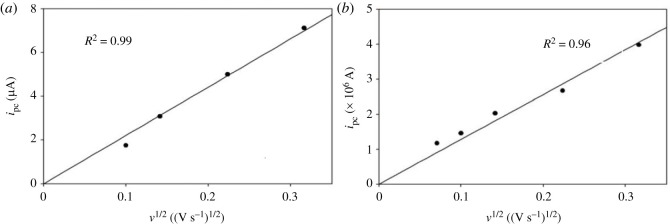
Variation of cathodic peak current with the square root of scan rate for the cyclic voltammograms of (*a*) 3 mM FeSO_4_ in conventional RME of TX-100 and (*b*) 20 mM FeSO_4_ in IL-based RME of TX-100 on a copper electrode at 30°C.

The effect of *v* on the reduction process of Fe^2+^ ions from these two RME systems was studied similarly as described for [C_2_mim]CF_3_SO_3_ medium (figure 2*a*). The linear relationship between *E*_pc_ and ln *ν* as shown in figure 6*a*,*b* for the electroreduction of Fe^2+^ in both of the RME systems indicates irreversible nature. The *i*_pc_ versus *ν*^1/2^ plots (figure 7*a*,*b*) for the electroreduction of Fe^2+^ in both of the RME systems were straight lines passing through the origin following equation (3.2). Thus, the electrochemical reduction of Fe^2+^ in conventional and IL-based RME systems used in the present study can be considered as irreversible diffusion-controlled process.

**Figure 8.**
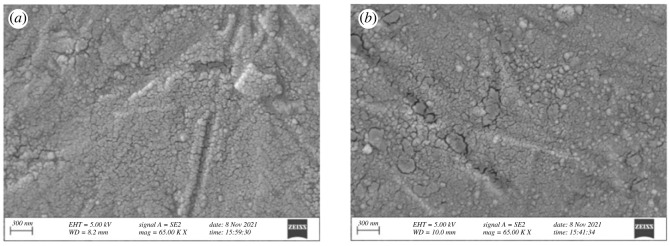
SEM images of electrodeposited Fe obtained by potentiostatic electrolysis from (*a*) 3 mM FeSO_4_ in conventional RME of TX-100 at −0.6 V for 15 min and (*b*) 20 mM FeSO_4_ in IL-based RME of TX-100 at −0.8 V for 30 min (temperature: 30°C).

Iron was electrodeposited from conventional RME containing 3 mM FeSO_4_ by bulk electrolysis for 15 min at –0.6 V. A compact deposition containing 40–50 nm spherical particles was obtained from the water-in-oil microemulsions as evident from the SEM image (figure 8*a*). Besides, the iron deposit obtained from [C_2_mim]CF_3_SO_3_ IL-based RME containing 20 mM FeSO_4_ at −0.8 V for 30 min on a copper substrate contained spherical particles of 30–40 nm in diameter (figure 8*b*).

**Figure 9.**
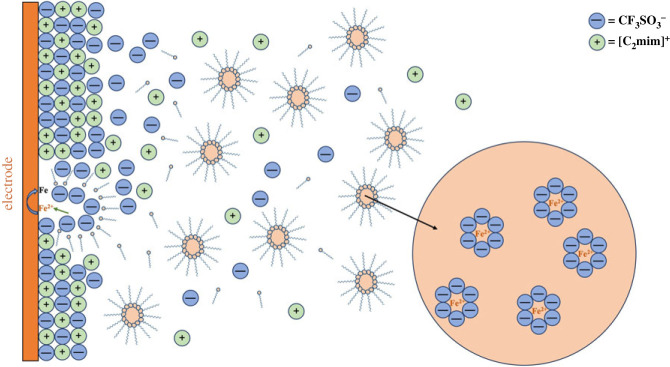
Simplistic representation of the mechanism of electrodeposition of iron from a bath of [C_2_mim]CF_3_SO_3_] IL-based RME of TX-100.

Figure 9 presents a simplified mechanism of electrodeposition of iron as speculated from a bath of IL-based RME of TX-100. In the conventional RME, solubilization of FeSO_4_ is supposed to take place inside the RME core containing polar water phase. Due to Brownian motion, the nano-sized RME cores [38] carrying the metal ions have continuous collisions with the electrode surface. Water cores inside the RME containing Fe^2+^ may be adsorbed on the electrode surface due to interaction between the electrode surface and RME cores. The adsorbed water cores containing Fe^2+^ can release the Fe^2+^, which will be reduced to form metallic iron. Thus, the Fe^2+^ ions are released in a controlled way which results in the slower deposition rate in RME. Moreover, the surfactant molecules are supposed to be adsorbed on the electrode surface, which can hinder the agglomeration of small nanoparticles and leads to the formation of granular nano-sized morphology [37].

In addition, the absence of ion crowding and thick double layer structure, which is known to form in IL systems near the electrode surface, the conventional RME systems leads to the formation of a highly desired compact deposition layer. Besides, along with the factors associated in the electrodeposition of metals in conventional RME, the viscosity of the IL and the metal–IL anion interaction are two important factors in the deposition process in IL-based RME. The viscosity of ILs is an important feature related to their liquid properties. Variation of viscosity of ILs can significantly affect their transport characteristics, such as diffusion coefficient, charge transfer, conductivity, etc. [48]. Therefore, due to higher viscosity of IL, the diffusion of Fe^2+^ ions from an IL core towards electrode surface is expected to be slower and more difficult than that from a water core. The cyclic voltammograms presented in figure 5 clearly show that the reduction current of Fe^2+^ ions in IL-based RME is lower than that of conventional RME indicating the IL-based RME system as a relatively viscous medium. Furthermore, the interaction of Fe^2+^ ion with the anion of the IL is obviously different from that of water as strong complex formation of metal ion with the anion of IL is well established. Therefore, in comparison with the conventional RME, the deposition process is supposed to be even slower and controlled in the IL-based RME as the solvated ions diffuse through a more viscous medium. However, it is worth mentioning that the IL-based RME system also contains a large amount of surfactant, which can also play a crucial role in the morphology of the deposit and a similar nucleation and crystal growth may occur as observed for conventional RME. Nevertheless, several factors, such as strong complexation of Fe^2+^ with the ions of IL, higher viscosity of the medium, electrostatic force of attraction between the ions in IL-based RME, and the unique double layer structure formed by the ions of IL near the working electrode surface, can improve the controllability of electroreduction of Fe^2+^ ions in IL-based RME.

## 4. Conclusion

In the present study, electrodeposition of metallic iron has been successfully achieved with tunable morphology and particle size from [C_2_mim]CF_3_SO_3_ and RME systems of TX-100. The electrochemical reduction process of Fe^2+^ was found to be irreversible and diffusion-controlled in IL, conventional RME, and IL-based RME systems. In the IL system, the presence of ion crowding and/or electric double layer structure near the electrode surface hindered the compact deposition of metallic iron. Interestingly, compact electrodeposition of iron could be achieved from RME systems. The controlled diffusion of Fe^2+^ towards electrode surface in both the conventional and IL-based RME systems leads to the deposition of compact granular nanoparticles. Moreover, the interaction of ions and higher viscosity of IL in IL-based RME can have additional impact on morphological aspects. It can be concluded that RME systems especially IL-based RME systems can be promising media to obtain compact deposition with tunable size and morphology.

## Data Availability

Data are available from the Dryad Digital Repository [49]. Supplementary material is available online [50].
